# Supplementary figures and images for: Study on geographic differentiation and environment-host synergistic assembly mechanism of root-associated fungal communities in Paphiopedilum purpuratum
Source: Microbiol Spectr. 2026 Feb 2;14(3):e02573-25. doi: 10.1128/spectrum.02573-25 (PMC12955379; doi:10.1128/spectrum.02573-25)

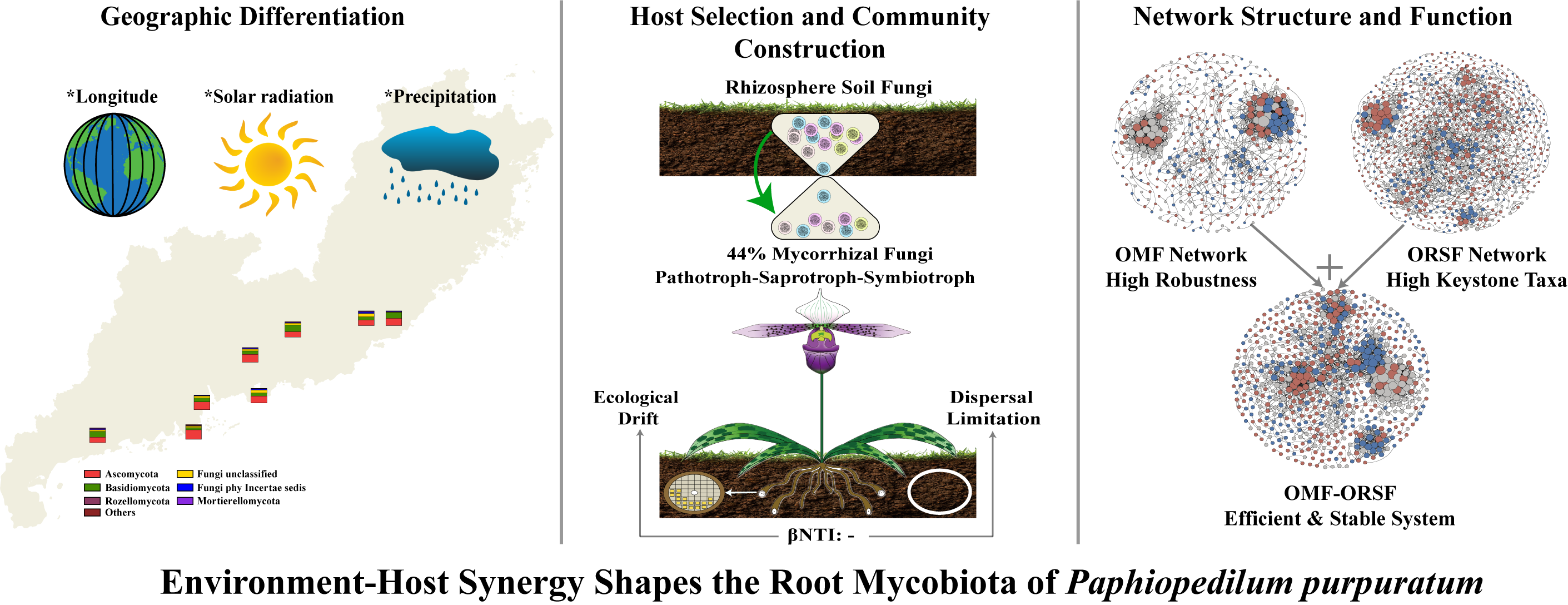

Supplement: GRAPHICAL ABSTRACT — For Paphiopedilum purpuratum, geographic environmental factors drive root mycobiota differentiation, while host selection (coupled with ecological drift and dispersal limitation) shapes its rhizosphere fungal community assembly—ultimately forming a robust, efficient OMF-ORSF network system via environment-host synergy. [file spectrum.02573-25-s0001.tif]
