# Supplementary material for: Study on geographic differentiation and environment-host synergistic assembly mechanism of root-associated fungal communities in Paphiopedilum purpuratum
Source: Microbiol Spectr. 2026 Feb 2;14(3):e02573-25. doi: 10.1128/spectrum.02573-25 (PMC12955379; doi:10.1128/spectrum.02573-25)
Supplement: Table S1 — Indices of the topological structure of the microbial community network. [file spectrum.02573-25-s0002.docx]

Table S1 Indices of the topological structure of the microbial community network

| Group | Node | Edge | Degree | BC | CC | EC | Abundance | MI | APL |
| --- | --- | --- | --- | --- | --- | --- | --- | --- | --- |
| OMF | 401 | 1485 | 7.406 | 265.377 | 0.146 | 0.071 | 0.144 | 0.717 | 5.338 |
| ORSF | 784 | 1749 | 4.462 | 1653.801 | 0.119 | 0.026 | 0.085 | 0.839 | 7.309 |
| OMF-ORSF | 565 | 2416 | 8.552 | 516.175 | 0.139 | 0.061 | 0.095 | 0.743 | 4.444 |

Core Node Metrics

Node: Fundamental unit representing individual microbial taxa or OTUs;

Edge: Statistical correlation or ecological connection between nodes;

Degree: Number of connections per node, visualized by node size, indicating species' interaction frequency;

Abundance: Average relative abundance of the microbial taxon, represented by node color intensity;

Network Structure Metrics

Modularity Index: Quantifies degree of network compartmentalization; higher values indicate more distinct functional modules;

Average Path Length: Mean shortest path between any two nodes; lower values suggest higher information transfer efficiency;

Betweenness Centrality: Measures a node's role as a network bridge; high-value nodes are crucial for maintaining connectivity;

Closeness Centrality: Reflects a node's proximity to all others; high-value nodes enable efficient network-wide propagation;

Eigenvector Centrality: Comprehensive measure of node importance incorporating neighbor significance; identifies core network hubs.
